# Supplementary material for: The Paracrine Role of Endothelial Cells in Bone Formation via CXCR4/SDF-1 Pathway
Source: Cells. 2020 May 26;9(6):1325. doi: 10.3390/cells9061325 (PMC7349013; doi:10.3390/cells9061325)
Supplement: Supplementary file 1 [file cells-09-01325-s001.pdf]

SUPPLEMENTARY FILE FOR CELLS JOURNAL

# The paracrine role of endothelial cells in bone formation via CXCR4/SDF-1 pathway

**Tal Tamari, Rawan Kawar-Jaraisy, Ofri Doppelt, Ben Giladi, Nadin Sabbah and Hadar Zigdon-Giladi**

## mRNA expression of angiogenic genes in EPCs

RNA was extracted from pellets of EPCs and buffy coat cells obtained from two donors; 1 µg RNA from each sample was taken to reverse transcription reaction. Next, cDNA was generated with High-Capacity cDNA Reverse Transcription Kit (Thermo Fisher Scientific, Waltham, MA, USA). Quantitative real-time polymerase chain reaction (qPCR) was performed using real time PCR (Biometra Analytik, Jena, Germany) and Fast SYBR™ Green Master Mix (Applied Biosystems™, Foster city, CA, USA). An angiogenic kit containing 96 angiogenic primers (angiogenesis H96, Bio Rad, 10025073) was used and the results normalized to generate fold change for each gene using the ΔΔCt method.

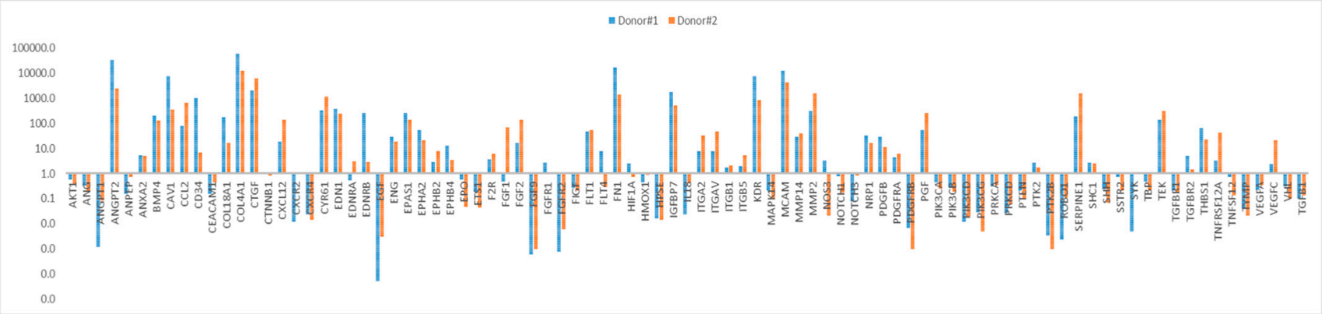


**Figure S1- mRNA expression of angiogenic genes in EPCs**

Relative quantification values of mRNA expression. Results were normalized relative to housekeeping gene and MNCs from the same donors.

## EPCs failed to differentiate into osteoblasts in-vitro

To investigate the osteogenic involvement of EPC in osteogenesis, we cultured the cells in an osteogenic differentiation medium. Osteogenic differentiation was evaluated using Alizarin Red staining (AR) and Alkaline Phosphatase staining (ALP) assays.

5x$10^3$ EPCs (passage 5) were seeded in 24-well plate and cultured in EGM-2 to attain 70-80 percent confluence. Then cells were grown for 21 days in osteogenic differentiation media containing EGM-2 with $10^{-7}$ M dexamethasone, $10^{-5}$ M ascorbic acid and $10^{-2}$ M β-glycerophosphate (Sigma Chemical Co., St. Louis, MO, USA). MSC served as control group and was cultured for the same time period in MSC standard medium (DMEM) supplemented with $10^{-7}$ M dexamethasone, $10^{-5}$ M ascorbic acid, and $10^{-2}$ M β-glycerophosphate . Osteogenic differentiation was identified by Alizarin Red (AR) staining (Sciencell Research Laboratories, Inc., California, USA) and alkaline phosphatase staining (Sigma Aldrich, California, USA).

According to the results, EPCs from two donors failed to differentiate to osteoblasts and didn't stain for AR (Fig 1A-2A) and ALP (1C-2C). However, MSCs (positive control cells) stained positive for AR (Fig. A) and ALP (Fig. C), indicating their ability to differentiate to osteoblasts.

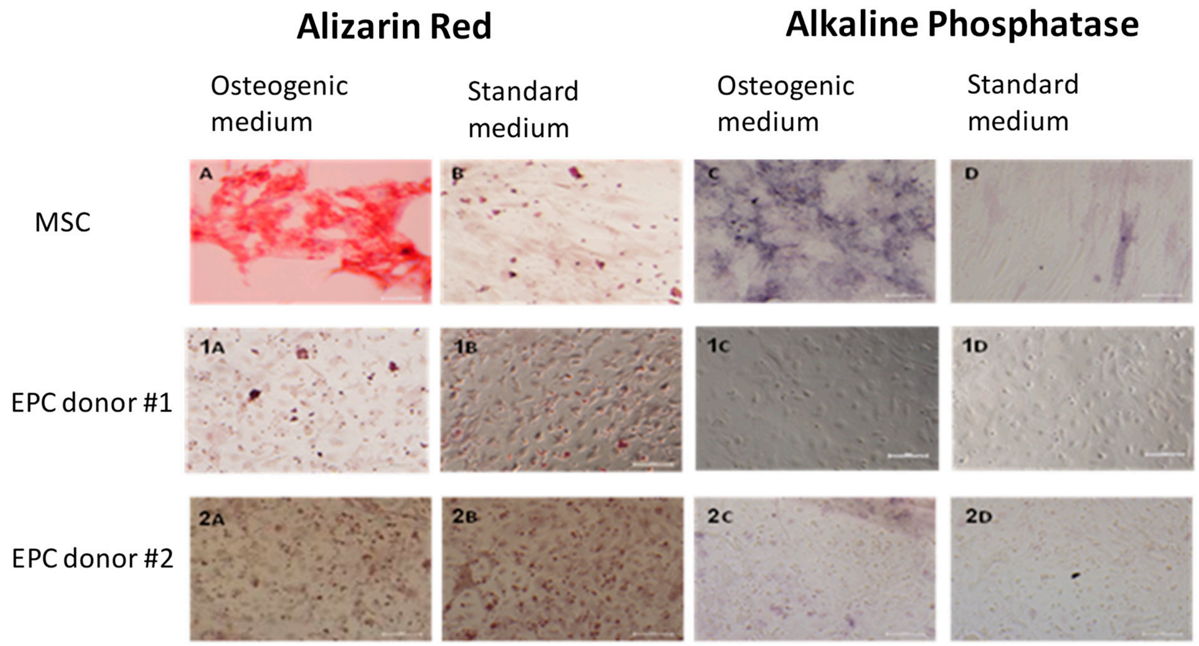


**Figure S2- Alizarin Red (AR) and Alkaline phosphatase (ALP) stainings following culture in osteogenic medium**

EPCs were cultured in osteogenic differentiation medium for 21 days and stained with AR (Fig. 1A-2A) and ALP (1C-2C). MSCs served as positive control, were positive for AR (Fig. A) and ALP (Fig. C).

## Migration of MSC and EPCs towards 5ng/ml C-C Motif Chemokine ligand 2 (CCL2)

Migration assay was performed with a 8 μm pore size millipore chamber (Millicell®, Darmstadt, Germany); 2.5 × $10^3$ MSCs or EPCs were seeded on the top of a porous membrane in 200 μL DMEM or EGM-2 (respectively) with 0.5% FBS. The lower chamber was filled with EGM-2, or EGM-2+ 5ng/ml CCL2 (PeproTech Asia Ltd., Rehovot, Israel). After overnight incubation, the cells were stained with crystal violet solution (Sigma-Aldrich) and the number of cells that migrated to the lower side of the membrane quantified. Results were less impressive compared to SDF-1. For this reason, we focused on SDF-1.

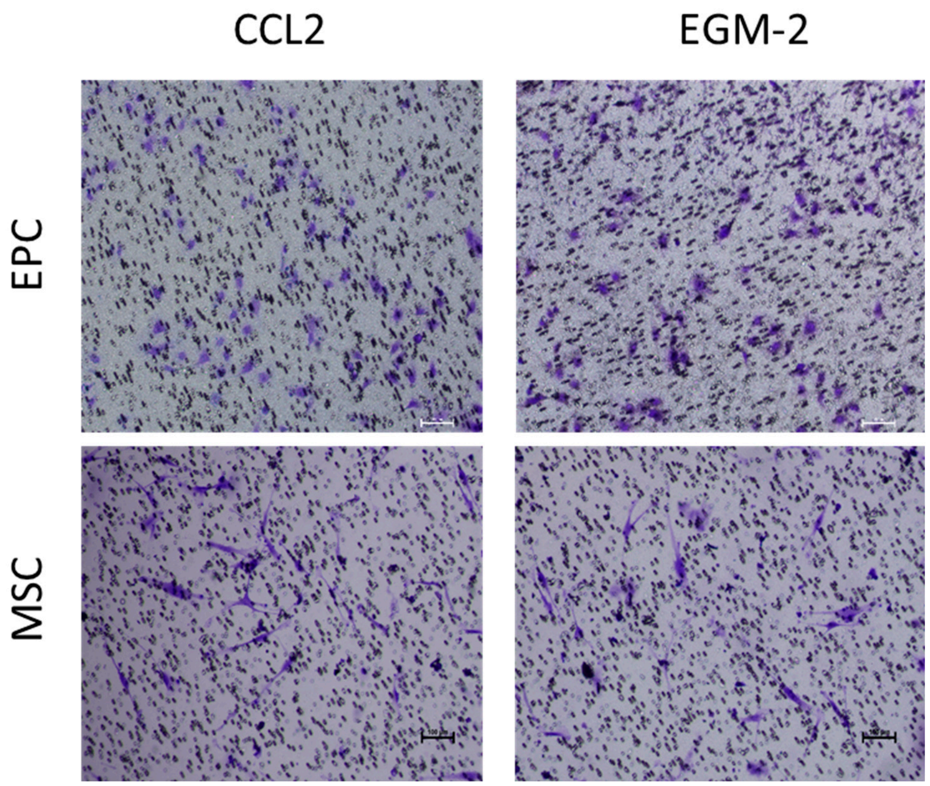


**Figure S3- Migration of EPC and MSC towards CCL2**

Migration of EPCs and MSCs towards CCL2 was evaluated in a transwell migration assay
